# Pretreatment of Rapeseed Meal Increases Its Recalcitrant Fiber Fermentation and Alters the Microbial Community in an *in vitro* Model of Swine Large Intestine

**DOI:** 10.3389/fmicb.2020.588264

**Published:** 2020-11-19

**Authors:** Cheng Long, Koen Venema

**Affiliations:** ^1^Faculty of Science and Engineering, Centre for Healthy Eating and Food Innovation, Maastricht University Campus Venlo, Venlo, Netherlands; ^2^School of Nutrition and Translational Research in Metabolism (NUTRIM), Maastricht University, Maastricht, Netherlands

**Keywords:** cell wall polysaccharides, rapeseed meal, pig gut microbiota, pectinase, cellulase

## Abstract

The aim of current study was to investigate in an *in vitro* study how enzymatic and chemical pretreated rapeseed meal (RSM) influences the fiber fermentation and microbial community in the swine large intestine. RSM was processed enzymatically by a cellulase (CELL), two pectinases (PECT), or chemically by an alkaline (ALK) treatment. 16S rRNA gene sequencing data was performed to evaluate changes in the gut microbiota composition, whereas short-chain fatty acid (SCFA) production (ion-chromatography) and non-starch polysaccharides (NSP) composition (using monoclonal antibodies; mAbs) were used to assess fiber degradation. The results showed that ALK, CELL, PECT1, and PECT2 changed microbial community composition, increased the predicted abundance of microbial fiber-degrading enzymes and pathways, and increased acetic acid, propionic acid, butyric acid, and total SCFA production. The increased microbial genera positively correlated with SCFA production. Monoclonal antibody analyses showed that the cell wall polysaccharide structures of RSM shifted after ALK, CELL, PECT1, and PECT2 treatment. The degradation of NSP during the fermentation period was dynamic, and not continuous based on the epitope recognition by mAbs. This study provides the first detailed analysis of changes in the swine intestinal microbiota due to RSM modified by ALK, CELL, PECT1, and PECT2, which altered the microbial community structure, shifted the predicted functional metagenomic profile and subsequently increased total SCFA production. Our findings that ALK, CELL, PECT1, and PECT2 increased fiber degradability in RSM could help guide feed additive strategies to improve efficiency and productivity in swine industry. The current study gave insight into how enzymatic treatment of feed can alter microbial communities, which provides good opportunity to develop novel carbohydrase treatments, particularly in swine feed.

## Introduction

Rapeseed meal (RSM), a by-product of rapeseed oil production, is not only a suitable protein source for swine feed but also a potentially energy source. RSM contains 20 to 40% non-starch polysaccharides (NSP) (Slominski and Campbell, 1990; Simbaya et al., 1995). The primary cell walls of RSM consist of pectin and xyloglucan and its cellulose microfibrils are interlinked with xyloglucan via hydrogen bonds forming a stiff network (Carpita and Gibeaut, 1993). Pectins are linked to each other and cross-linked between pectin and hemicellulose, and between pectin and cellulose (Broxterman and Schols, 2018). Pectins, consisting of homogalacturonan, rhamnogalacturonan, xylogalacturonan, arabinogalactan and arabinan, are the major polysaccharides present in the dehulled rapeseed meal (Eriksson et al., 1997). In the secondary cell wall of RSM, the main carbohydrates are 4-O-methyglucuronoxylan, xyloglucan, and cellulose. A drawback of using RSM in animal feed is that the complex cell wall polysaccharides cannot be utilized by endogenous enzymes from monogastric animals (e.g., pigs), and also can only partly be fermented by the microbial community in the gastrointestinal tract (GIT) of the pig.

Therefore, the animal feed sector seeks opportunities to enhance degradability of NSP of feedstuffs, in order to improve its potential as a nutrient source for domestic animals. Previous research showed that physical processing technologies, such as hammer milling, pelleting, wet-milling, extrusion, and mild hydrothermal acid treatment, had limited effect on recalcitrant NSP structures (de Vries et al., 2012, 2013). As a result, more efficient solutions are needed to modify the cell wall architecture and allow the gut microbiota to utilize the complex carbohydrates. Former research has shown that NSP-degrading enzymes, such as cellulase and pectinases, could open the cell wall structure and improve NSP degradability (Giraldo et al., 2007, 2008). Previous studies showed that addition of pectolytic enzymes improved degradability of NSP of RSM *in vitro* (Pustjens et al., 2012) and in broilers (de Vries et al., 2014; Pustjens et al., 2014). However, none of the above studies investigated how the gut microbiota was affected by the modified RSM or by the feed enzymes. It is important to know this, as NSP can only be fermented by microbes. Bindelle et al. (2011) demonstrated that NSP-degrading enzymes increased abundances of cellulolytic *Ruminococcus*- and xylanolytic *Clostridium*-like bacteria and altered fermentation patterns of barley cultivars and wheat products. Torok and colleagues (Torok et al., 2008) investigated changes in gut microbial population in response to the supplementation of an NSP-degrading enzyme (containing β-glucanase, xylanase, and protease activities) in a barley-based diet in chickens, and the results showed that microbial composition revealed distinct clusters correlating with un-supplemented and enzyme supplemented birds. Previous research reported that the pre-treatment of feed stuffs with carbohydrases can cause the release of reducing sugars and other hydrolysis materials, promoting chemotactic response in specific bacteria, and stimulating their attachment to feed particles, and thereby growth of these microbes (Beauchemin et al., 2003; Giraldo et al., 2007; Ribeiro et al., 2015, 2018).

In the present study, RSM (predigested with digestive enzymes) was treated independently with two kinds of pectinases (PECT1 and PECT2), one cellulase (CELL), or alkaline (ALK), and afterwards the untreated and treated RSM preparations were fermented in the Swine Large Intestine *in vitro* Model (SLIM) (Long et al., 2020a). The aim of the current study was to investigate whether fermentation by the swine gut microbiota of treated RSM was improved compared to untreated RSM, and whether the microbiota composition and activity were changed.

## Materials and Methods

### Substrate Preparation

**FIGURE 1:**
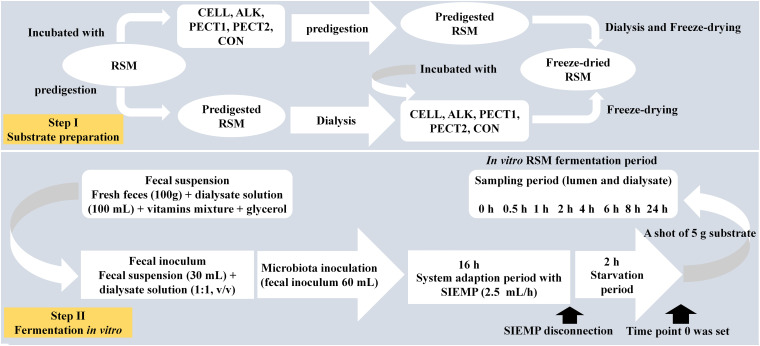
Schematic experimental setup for fiber fermentation in the Swine Large Intestine *in vitro* model (SLIM).

Rapeseed meal (Brassica napus, Cargill N.V., Antwerp, Belgium; 2011) was obtained from a commercial feed mill (Agrifirm B.V., Utrecht, Netherlands). Preparation method I (predigestion of RSM after carbohydrase or alkaline treatment) (Figure 1): to 200 g of RSM 40 mL 10^∗^gastric electrolyte concentrate solution (GES, 310 g sodium chloride, 110 g potassium chloride, 15 g calcium chloride di-hydrate, and 4840 g ultrapure water) and 360 mL ultrapure water were added. The pH was adjusted to 5.5 and then nothing (CON), 10 mL of alkaline (ALK, 6 M NaOH), or the following carbohydrases were added CELL (Accellerase 1000, Sigma-Aldrich, Missouri, United States), PECT1 (Pectinex Ultra SP, Novozymes A/S, Bagsvaerd, Denmark), or PECT2 (Multifect Pectinase, DuPont Industrial Biosciences, Genencor division, Rochester, NY). CELL has cellobiohydrolases, endoglucanases, beta-glucosidases, and hemi-cellulases activities. PECT1 has pectin esterase and polygalacturonase activities. PECT2 mainly has pectinase and some hemicellulase activities. Enzyme preparations were added to the RSM (25 mL/kg DM) and incubated at 37°C for 2 h, with occasional shaking (every 30 min), while ALK was incubated overnight at 4°C. Enzyme preparations were then heated at 100°C for 5 min to inactive enzymes. Afterwards, for all five samples, 120 mL GES was added and pH adjusted to 3 to continue with the gastric incubation according to the predigestion protocol as described elsewhere (Sáyago-Ayerdi et al., 2017). After predigestion, the slurry was centrifuged (8.000 *g*, at 4°C, for 20 min) and dialysis was performed for the supernatants. For dialysis, a dialyzer (Sureflux, Nipro Europe Group Companies, Mechelen, Belgium) was used with a peristaltic pump to remove small digestion products and water. After reduction of the total volume to ∼450–500 mL, supernatant was mixed with pellet, and freeze-dried. Method II (predigestion of RSM before carbohydrase or alkaline treatment) (Figure 1): four batches of 200 g RSM were predigested as described before (Sáyago-Ayerdi et al., 2017) and then dialyzed. Afterwards, 55 mL 10^∗^GES was added, and pH adjusted to 5.5, after which 10 mL of ALK, or 10 mL of CELL, PECT1, or PECT2 treatment (25 mL/kg DM) commenced, respectively. Enzyme preparations were incubated at 37°C for 2 h with occasional shaking (every 30 min), and ALK was incubated overnight at 4°C. Afterwards, enzyme preparations were heated at 100°C for 5 min to inactive enzymes, and pH was neutralized to 6.5–7 with HCl or NaOH, and the samples were freeze-dried. Samples are differentiated by the suffix _B (for before) or _A (for after) (e.g., PECT1_A) for carbohydrase- or ALK-treatment prior to and after digestion, respectively.

### Fermentation in the Swine *in vitro* Large Intestinal Model (SLIM)

The setup of SLIM was as follows: a fully computer-controlled *in vitro* model based on TIM-2 (Minekus et al., 1999) was used to mimic the swine large intestine (Long et al., 2020a). The pH (5.9) was controlled by continuous addition of 2 M sodium hydroxide. Standard ileal efflux medium of pigs (SIEMP) was used to simulate the materials entering the colon. The SIEMP, adapted from Gibson et al. (1988) and described in Long et al. (2020a), contained the following components (g/L): 74.6 maize starch, 9.0 xylan, 19.0 pectin, 9.0 amylopectin, 9.0 arabinogalactan, 9.0 arabinoxylan, 9.0 xyloglucan, 31.5 Tween 80, 43.7 casein, 0.7 ox-bile, 43.7 bactopepton, 4.7 K_2_HPO_4_.3H_2_O, 0.009 FeSO_4_.7H_2_O, 8.4 NaCl, 0.8 CaCl_2_.2H_2_O, 0.7 MgSO_4_.7H_2_O, 0.05 bile, 0.02 haemin and 0.3 cysteine∘HCl, plus 1.5 mL of a vitamin mixture containing (per liter): 1 mg menadione, 0.5 mg vitamin B12, 2 mg D-biotin, 10 mg pantothenate, 5 mg p-aminobenzoic, 4 mg thiamine and 5 mg nicotinamide acid. The pH was adjusted to 5.9. Dialysis liquid contained (per liter): 2.5 g K_2_HPO_4_.3H_2_O, 0.005 g FeSO_4_.7H_2_O, 4.5 g NaCl, 0.45 g CaCl_2_.2H_2_O, 0.05 g bile, 0.5 g MgSO_4_.7H_2_O and 0.4 g cysteine∘HCl, plus 1 mL of the vitamin mixture. All medium components were purchased at Tritium Microbiology (Eindhoven, Netherlands). The pig fecal inoculum was a standardized microbiota from growing pigs, freshly collected from the floor (48 pens with 6 pigs/pen, Hypor Libra x Hypor Maxter, Hendrix Genetics, Boxmeer, Netherlands), but only material from the top was selected (so not toughing the floor). Feces was pooled and mixed with dialysate as described before (Long et al., 2020a).

In order to create a complete anaerobic environment, SLIM with 90 mL dialysate in each of the 4 individual units was flushed with gaseous nitrogen for at least 3 h before incorporating the standardized microbiota. Thirty mL of the standardized microbiota was added to each SLIM-unit, making the total volume 120 mL. Figure 1 shows the experimental set-up for fiber addition to SLIM. The microbiota was adapted to the model with SIEMP for 16 h. During the adaptation phase, SIEMP was added into each SLIM-unit at a rate of 2.5 mL/h through the feeding syringe. At the end of the adaptation period, a 2-h starvation period was performed, which was used to allow all the carbohydrates within SIEMP to be fermented. Afterwards, a shot of 5 grams of the different RSMs was given to the system at time point 0 h, and incubation was continued for 24 h after that (Figure 1).

### Sample Collection

Samples from lumen (*n* = 2) and spent dialysate (*n* = 2) were collected at time point 0, 0.5, 1, 2, 4, 6, 8, and 24 h (t0, t0.5, t1, t2, t4, t6, t8, and t24). They were snap-frozen in liquid nitrogen and stored until analyses. Lumen samples were used to analyze microbiota composition and polysaccharides structures, and both lumen and dialysis samples were analyzed for short chain fatty acid (SCFA) concentrations.

### Sequencing of the V3-V4 Region of the 16S rRNA Gene

Microbial DNA extraction and sequencing of the V3-V4 region of the 16S rRNA gene were performed by BaseClear B.V. (Leiden, Netherlands). Briefly, genomic DNA extraction was performed using the Quick-DNA^TM^ Fecal/Soil Microbe Miniprep Kit (Zymo Research, California, United States) according to the manufacturer’s instructions. Barcoded amplicons from the V3-V4 region of 16S rRNA genes were generated using a 2-step PCR. 10–25 ng genomic DNA was used as template for the first PCR with a total volume of 50 μL using the 341F (5′-CCTACGGGNGGCWGCAG-3′) and the 785R (5′-GACTACHVGGGTATCTAATCC-3′) primers (Klindworth et al., 2013) appended with Illumina adaptor sequences. PCR products were purified (QIAquick PCR Purification Kit, Venlo, Netherlands) and the size of the PCR products were checked on a Fragment analyzer (Advanced Analytical, Ankeny, United States) and quantified by fluorometric analysis. Purified PCR products were used for the 2nd PCR in combination with sample-specific barcoded primers (Nextera XT index kit, Illumina, CA, United States). Subsequently, PCR products were purified, checked on a Fragment analyzer and quantified, followed by multiplexing, clustering, and sequencing on an Illumina MiSeq with the paired-end (2×) 300 bp protocol and indexing. The sequencing run was analyzed with the Illumina CASAVA pipeline (v1.8.3) and demultiplexed based on sample-specific barcodes.

### Bioinformatics Analysis

The demultiplexed raw sequences obtained from BaseClear were processed using the QIIME2 pipeline (Bolyen et al., 2019). In short, reads were imported and quality filtered and dereplicated with q2-dada2 (Callahan et al., 2016). Next, dada2 was performed with paired-end reads and truncations parameters were as follows:the first 17 and 14 base pairs were trimmed off in forward and reverse reads, respectively. And at position 280 base pairs the fragment was truncated in forward reads, and at position 230 base pairs for the reverse reads. The processed sequences were used for all the downstream analyses. Alpha-diversity (Shannon index) and β-diversity (weighted and unweighted UniFrac) were analyzed by the q2-phylogeny plugin^1^.

### Phylogenetic Investigation of Communities by Reconstruction of Unobserved States, PICRUSt2

The PICRUSt2 software (Douglas et al., 2019) was used to predict microbial functional abundances based on marker gene sequences. KEGG database was used to predict the results.

### Chemical Analyses

#### Short-Chain Fatty Acids Analyses

Samples from lumen and dialysate were analyzed by Brightlabs (Venlo, Netherlands) for determination of concentrations of SCFA. Ion exclusion chromatography (IEC) was applied on an 883 Ion Chromatograph (IC; Metrohm, Switzerland), using a Transgenomic IC Sep ICE-ION-300 column (30 cm length, 7.8 mm diameter, and 7 μm particles) and a MetroSep RP2 Guard. The mobile phase consists of 1.5 mM aqueous sulphuric acid. Samples were centrifuged (21,000 *g*, 10 min) and the clear supernatant was filtered through a 0.45 μm PFTE filter and diluted with mobile phase (for lumen 1:5, for dialysate 1:2). Ten microliters were loaded on the column by an autosampler 730 (Metrohm). Molecules were eluted according to their pKa.A column flow rate of 0.4 ml^∗^min^–1^ was used. The temperature of the column was 65°C. The organic acids were detected using suppressed conductivity detection.

#### Glycome Profiling

##### Sample preparation

Lumen samples from each time point and treatment were freeze-dried, after which they were dissolved at 1 mg/mL in deionized water, and stored at −20°C as stock solutions.

##### Monoclonal antibodies (mAbs)

mAbs were obtained as hybridoma cell culture supernatants from CarboSource (Atlanta, GA, United States)^2^ (Supplementary File 1).

##### ELISA

The ELISA protocol was slightly modified from Pattathil et al. (2010). In brief, samples prepared above were applied (50 μL of 100 μg/mL in deionized water per well) to Costar 3598 96-well plates (Corning Life Sciences, New Yok, United States) and were dried to the well surfaces by evaporation overnight at 37°C. Control wells contained deionized water. The plates were blocked with 200 μL of 1% (w/v) bovine serum albumin (BSA) in Tris-buffered saline (50 mM Tris–HCl, pH 7.6, containing 100 mM sodium chloride) for 1 h. Blocking agent was removed by aspiration, and 50 μL of undiluted hybridoma supernatant were added to each well and incubated for 1 h at room temperature. Supernatant was removed and wells were washed three times with 200 μL of 0.1% (w/v) BSA in Tris-buffered saline (wash buffer). Peroxidase-conjugated goat anti-mouse IgG, anti-mouse IgM, goat anti-rat IgG, or goat anti-rat IgM antibodies (Sigma-Aldrich), depending on the primary antibody used, were diluted 1:5,000 in wash buffer, and 50 μL were added to each well and incubated for 1 h. Wells were then washed five times with 200 μL of wash buffer. Next, 3,3′,5,5′-tetramethylbenzidine (TMB) solution (Sigma-Aldrich, St. Louis, United States) was freshly prepared according to the manufacturer’s instructions, and 50 μL were added to each well. After 20 min, the reaction was stopped by adding 50 μl of 0.5 N sulfuric acid to each well. The OD of each well was read at a wavelength of 450 nm using a Multi-mode microplate reader (BioTek Synergy HTX, Abcoude, Netherlands).

##### Polysaccharide panel screening

Polysaccharide panel screening of mAbs was carried out by ELISA against all lumen samples immobilized to 96-well plates. Duplicate preparations of each polysaccharide were used for all experiments reported here. Binding data was visualized in R via ComplexHeatmap package (Gu et al., 2016).

### Statistics

Analysis of covariance (ANCOVA) was applied to compare α-diversities (Shannon index), relative abundances of taxa at genus level and predicted abundance of microbial functional profile among different RSM treatments and time points by using package lme4 in R version 3.5.3^3^. Visualizations were performed in STAMP (Parks et al., 2014). The amplicon sequence variant (ASV) table (feature table of QIIME2) was normalized and filtered in R. The table was normalized via division by the sum of sequences in a given sample and multiplied by the minimum sum across all samples. Relative abundances were filtered as follows: values below a relative abundance threshold of 0.01% were not taken into account; taxa with a median relative abundance <1% in all groups were not considered for statistical analysis. White’s non-parametric *t*-test was applied to compare between CON and treatments.

Permutational multivariate analysis of variance [PERMANOVA (Anderson, 2005)] was performed to test the significance of β-diversity distances in QIIME2 (weighted and unweighted UniFrac) between non-processed and processed RSM. The results were visualized in R (R version 3.5.3).

Pearson correlations between continuous meta-variables and taxonomic variables were calculated and visualized in R. Parameters were set as follows: Missing values for meta-variables were handled as NO imputation (replacing missing data with substituted); zeros were kept for the calculation of correlation; a minimum number of 0.1% was considered for calculation; a minimum of 4 paired observations were required for calculation of correlations. *P*-values were corrected using the Benjamini-Hochberg method. A *q*-value (corrected *P*-value) <0.05 was considered significant.

## Results

### Characteristics of Non-processed and Processed RSM

A comprehensive set of 155 plant cell wall glycan-directed monoclonal antibodies was used to screen untreated RSM by a ELISA-based assay (Pattathil et al., 2010, 2012), and 34 antibodies reacted with RSM (data not shown). These were subsequently used in the current study to obtain information on the presence and relative abundance of specific epitopes that are characteristic of the different types of polymers in untreated RSM and RSM processed by ALK, CELL, PECT1, and PECT2.

**FIGURE 2:**
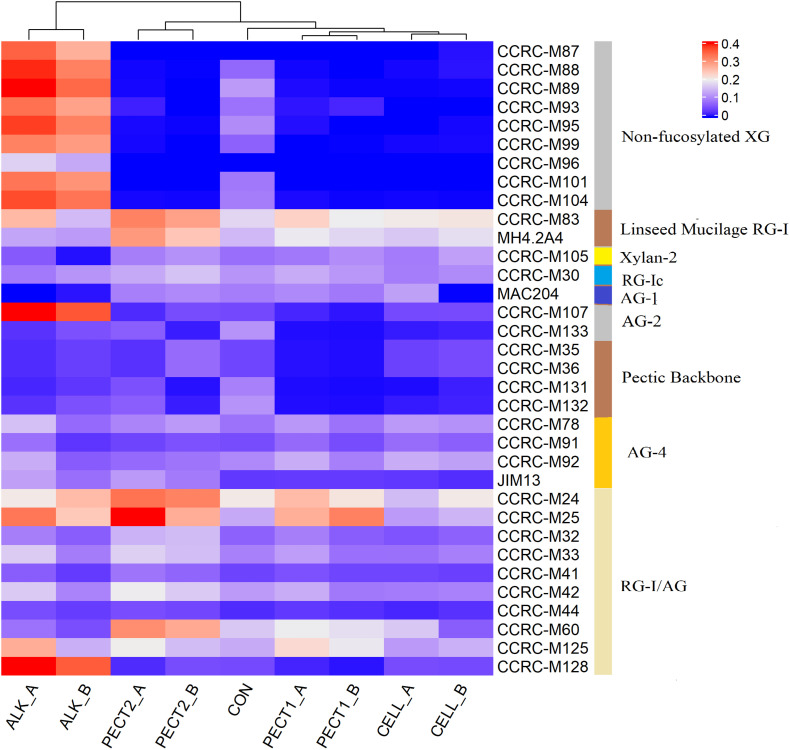
Glycome profiling of non-processed and processed RSM. The binding response data of the mAbs are presented as heatmap using a blue-white-red scale indicating the intensity of the ELISA signal (blue, white, and red colors depict no, medium, and strong binding, respectively). The mAbs (indicated by their codes) are grouped based on the cell wall glycans they predominantly recognize as shown in the panel on right-hand side of the figure. _A, RSM was treated after predigestion, _B; RSM was treated before predigestion. XG, xyloglucan; RG-I, rhamnogalacturonan I; AG, arabinogalactan; RG-I/AG, arabinogalactan side chains of RG-I.

Figure 2 shows that both increases and decreases in epitope recognition occurred in ALK, CELL, PECT1, and PECT2 compared to CON. Samples from after (_A) and before (_B) predigestion clustered together according to each treatment, which indicated _A and _B from the same treatment had similar epitope accessibility. ALK strongly increased binding of non-fucosylated XG mAbs, while CELL, PECT1, and PECT2 led to disappearance of those compared to CON, regardless of _A and _B treatment. All the treatments increased the binding of “Linseed Mucilage RG-I group” directed mAbs, but had little effect on Xylan-2 and RG-Ic group compared to CON. Binding of MAC204 (AG-1), which is binding to gum tragacanth and to lettuce and green tomato RG-I preparations (arabinogalactan), disappeared with ALK_A, ALK_B, and CELL_B, while increased binding of CCRC-M107 (AG-2), which binds to linear and branched arabinans and RG-I preparations from diverse plants but does not bind to larch arabinogalactan (Pattathil et al., 2010), was observed in ALK_A and ALK_B. CELL_A, CELL_B, PECT1_A, PECT1_B, and PECT2_B led to disappearance of the binding of mAbs of “pectic backbone group” and CCRC-M 133, which also binds to linear and branched arabinans and RG-I preparations from diverse plants but do not bind to larch arabinogalactan. ALK, PECT1, and PECT2 increased binding of mAbs directed against the arabinogalactan side chains of RG-I (RG-I/AG).

### ALK, CELL, PECT1, and PECT2 Significantly Changed Microbiota Composition Compared to CON as Evaluated by Unweighted UniFrac

**FIGURE 3:**
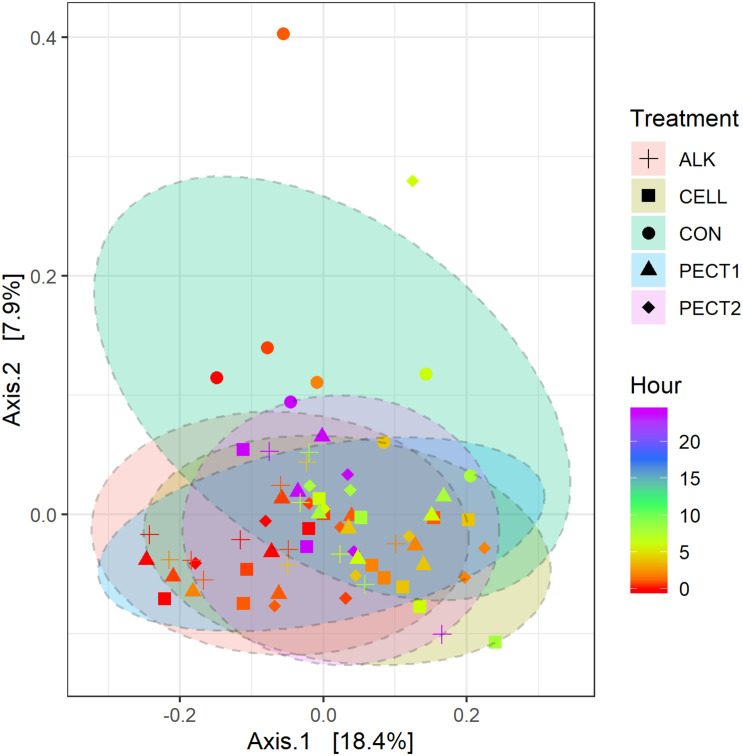
Principal coordinate analysis (PCoA) plot generated based on an unweighted UniFrac matrix. Samples were grouped by shape and color representing the treatment and time point they belonged to, respectively: CON (sphere), ALK (plus), PECT1 (triangle), PECT2 (diamond), CELL (square); a red-green-purple scale was used to indicate the fermentation time (red and purple depict start and end of the fermentation period).

To determine the changes in composition of the gut microbiota fed with a shot of 5 g CON, ALK, CELL, PECT1, or PECT2, a comparison of microbiota based on sequencing the V3-V4 region of the 16S rRNA gene was performed. Shannon indices significantly decreased at t4, t6, t8 and t24, compared to that of t0 (Supplementary File 2 and Supplementary Figure S1A). When data from all of the time points were pooled, there were no significant differences among CON, ALK, CELL, PECT1, and PECT2 in Shannon index (Supplementary File 2 and Supplementary Figure S1B). Phylogeny based UniFrac methodology was then used to compare the β-diversity of the microbial communities between microbiota fed with non-processed and processed RSM. Unweighted UniFrac analysis (Figure 3) shows that samples from processed RSM (ALK, CELL, PECT1, and PECT2) significantly (*P* = 0.004) separated from non-processed RSM (CON), and samples from different processing method clustered together. Samples from CON, ALK, CELL, PECT1, and PECT2 all clustered together (*P* = 0.125) with respect to weighted UniFrac (Supplementary File 2 and Supplementary Figure S2).

There were no significant differences between microbiota fed with RSM predigested before or after carbohydrase or ALK treatment with respect to both α-diversity (Supplementary File 2 and Supplementary Figure S3), or β-diversity (data not shown), which indicated method I and method II of processing RSM had little effect on microbiota composition. Glycome profiling of RSM (Figure 2) also shows that samples from after and before processing clustered together according to each treatment, which indicated their polysaccharide compositions were similar to each other.

**FIGURE 4:**
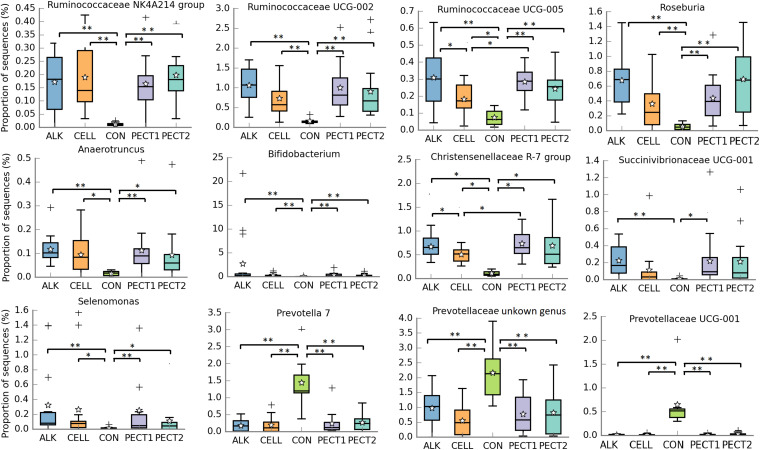
Significantly different relative abundances of microbial genera upon treatment with ALK, CELL, CON, PECT1, and PECT2. The relative abundances of genera in pre-treated RSM compared to CON were analyzed by ANCOVA. *P < 0.05; **P < 0.01. The stars represent the group means while the middle bars of the boxplots represent the median and the plusses represent outliers.

Relative abundances of taxa within the pig microbiotas fed with non-processed and processed RSM were compared to identify significantly different bacterial taxa. At genus level (Figure 4), seven genera were significant higher in relative abundance after ALK, CELL, PECT1, and PECT2 treatment compared to CON. These were *Ruminococcaceae NK4A214* group, *Ruminococcaceae UCG*-*002*, *Ruminococcaceae UCG*-*005*, *Roseburia*, *Anaerotruncus*, *Bifidobacterium*, *Christensenellaceae R-7* group, and *Selenomonas*. For genera *Christensenellaceae R-7* group and *Ruminococcaceae UCG-005*, their relative abundances were also higher in ALK and PECT1 compared to CELL. Instead, the relative abundances of *Prevotella* 7, an unclassified genus from *Prevotellaceae*, and *Prevotellaceae* UCG-001 were significantly decreased after feeding ALK, CELL, PECT1, and PECT2 compared to microbiota fed with CON. The relative abundance of *Succinivibrionaceae UCG-001* was significant higher in ALK and PECT1 compared to CON.

### PICRUSt2 Analyses Revealed That Microbial Functional Abundances Related to Carbohydrate Metabolism and SCFA Production Were Significantly Increased With Processed RSM Compared to CON

**FIGURE 5:**
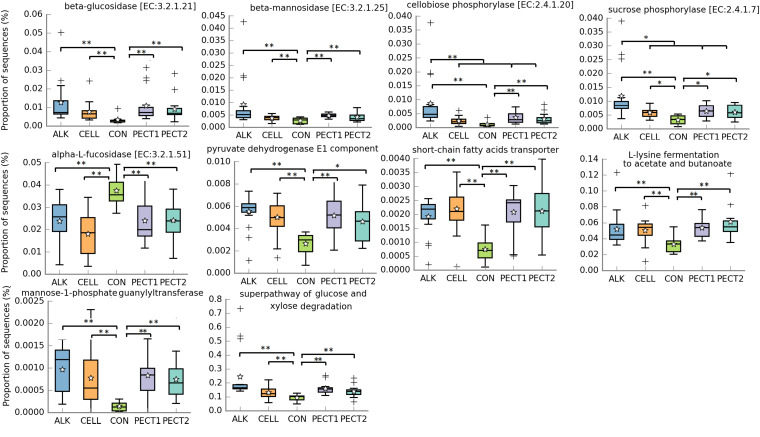
Significantly different metagenomic functions in relative abundance among ALK, CELL, CON, PECT1, and PECT2. The predicted abundances of microbial functional profile in pre-treated RSM compared to CON were analyzed by ANCOVA. *P < 0.05; **P < 0.01. The stars represent the group means while the middle bars of the boxplots represent the median and the plusses represent outliers.

PICRUSt2 was performed to the 16S rRNA gene data to predict metagenomic functional profiles. In this study we focused on carbohydrate metabolism related microbial functions (Figure 5). The relative abundances of fiber degradation pathways, beta-glucosidase [EC:3.2.1.21], beta-mannosidase [EC:3.2.1.25], and cellobiose phosphorylase [EC:2.4.1.20], were significant higher in ALK, CELL, PECT1, and PECT2 compared to CON, whereas conversely that of alpha-L-fucosidase [EC:3.2.1.51] was significant higher in CON compared to ALK, CELL, PECT1, and PECT2. For cellobiose phosphorylase [EC:2.4.1.20], the relative abundance in ALK was also significant higher than those of CELL, PECT1, and PECT2.

Six microbial pathways related to fermentation were significant higher in relative abundance when microbiotas were fed with ALK, CELL, PECT1, and PECT2 compared to microbiota fed with CON. These pathways were pyruvate dehydrogenase E1 component, short-chain fatty acids transporter, mannose-1-phosphate guanylyltransferase, superpathway of glucose and xylose degradation, and L-lysine fermentation to acetate and butanoate. The relative abundance of lactose/L-arabinose transport system permease protein was significant higher in ALK, PECT1, and PECT2 compared to CON.

Table 1 shows that the cumulative acetic, propionic, and butyric acid and total SCFA production were higher in ALK, CELL, PECT1, and PECT2 compared to CON. For acetic acid, more than 2 times greater production was observed when the microbiota was fed with ALK, CELL, PECT1, and PECT2 compared to when the microbiota was fed with CON. The production of propionic, butyric acid and total SCFA in ALK, CELL, PECT1, and PECT2 were more than 1.6 times higher than that in CON, except for propionic (1.3 times), and butyric acid (1.4 times) production in ALK.

**TABLE 1:** Fold change of cumulative Acetic, Propionic, and Butyric acid, and total short-chain fatty acid production during fermentation of ALK, PECT1, PECT2, and CELL compared to CON.

Treatment	Acetic acid	Propionic acid	Butyric acid	Total SCFA
ALK	2.0	1.3	1.6	1.4
CELL	2.3	1.7	1.8	1.7
PECT1	2.2	1.6	1.6	1.6
PECT2	2.5	1.9	1.8	1.8

### Glycome Profiling Shows That Binding of mAbs in Digests Were Dynamic During the *in vitro* Fermentation in SLIM

**FIGURE 6:**
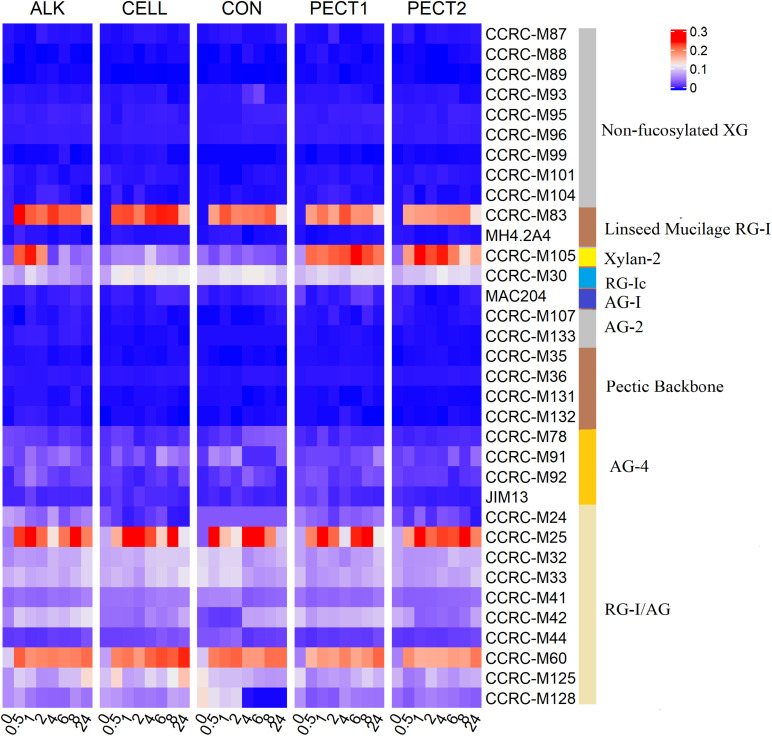
Glycome profiling of lumen digests during *in vitro* fermentation of ALK, CELL, CON, PECT1, and PECT2 at time point 0, 0.5, 1, 2, 4, 6, 8, and 24 h. The binding response data are presented as heatmap using a blue-white-red scale indicating the strength of the ELISA signal (blue, white, and red colors depict no, medium, and strong binding, respectively). The mAbs are grouped based on the cell wall glycans they predominantly recognize as shown in the panel on the right-hand side of the figure. XG, xyloglucan; RG-I, rhamnogalacturonan I; AG, arabinogalactan; RG-I/AG, arabinogalactan side chains of RG-I.

To investigate the dynamic changes of polysaccharides structure in CON, ALK, CELL, PECT1, and PECT2 during *in vitro* fermentation, a time series of sampling was performed and the set of 34 mAbs was used to screen the lumen digests. Figure 6 shows that no or few binding signals were observed in Non-fucosylated XG, AG-2, and Pectic Backbone mAbs upon feeding CON, ALK, CELL, PECT1, and PECT2 at all time points.

No binding of CCRC-M83 that specifically bind to Linseed Mucilage RG-I was observed at t0 in all treatments (just prior to addition of the fiber shots), and binding signals appeared afterwards. For ALK, binding of CCRC-M83 decreased from t0.5 to t2, increased again at t4, and decreased to the lowest value at t24. For CELL, binding of CCRC-M83 decreased from t0.5 to t2, stabilized from t4 to t8, and decreased to the lowest value at t24. For CON, binding increased from t0.5 to t1, decreased from t2 to t6, increased at t8, and decreased to the lowest value at t24. For PECT1, binding increased from t0.5 to t4, and then decreased progressively until t24. For PECT2, binding increased slightly from t0.5 to t8, and then decreased to the lowest value at t24.

In terms of Xylan-2 recognizing mAb (i.e., CCRC-M105), weak bindings were detected at all time points after t0 with CELL and CON. Increased binding of CCRC-M105 was observed from t0.5 to t1 in ALK and the amount of binding decreased from t2 to t4, slightly increased again at t6, and then decreased until t24. For PECT1, increased binding of CCRC-M105 was observed from t0.5 to t6, which decreased afterward until t24. Binding for PECT2 was dynamic from t0.5 to t24, but the lowest binding was observed at t24.

As for the RG-Ic recognizing mAb (i.e., CCRC-M30), binding of CCRC-M30 was lower in ALK, PECT1, and PECT2 according to each time point compared to CELL and CON, but binding over time was dynamic. With respect to the AG-4 mAbs (recognizing arabinogalactans), weak and dynamic binding was observed at each time point in all treatments.

With respect to RG-I/AG mAbs, more active mAbs were observed compared to other groups of mAbs in all treatments. Within RG-I/AG mAbs, amount of binding of CCRC-M25 and CCRC-M60 were stronger than other RG-I/AG mAbs in all treatments, and their amount of binding were fluctuating during the whole fermentation period and still existed at t24 in all treatments.

### Correlation Between Microbiota Abundance and SCFA Production and mAb Binding

**FIGURE 7:**
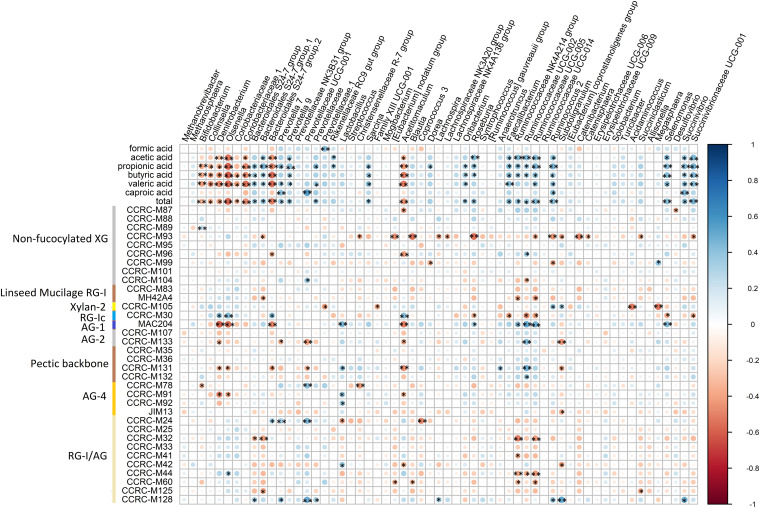
Correlation between core bacterial genera, and SCFA and binding of mAbs. Statistical significance was determined for all pairwise comparisons using Pearson’s method. *P < 0.05; **P < 0.01. Size of the circles indicates correlation strength, which bigger sizes meaning higher correlations. Blue circles represent positive correlations (correlation coefficients from 0 to 1), whereas red circles represent negative correlations (correlation coefficients from 0 to –1). total, total SCFA production. The mAbs are grouped based on the cell wall glycans they predominantly recognize as shown in the panel on the left-hand side of the figure. XG, xyloglucan; RG-I, rhamnogalacturonan I; AG, arabinogalactan; RG-I/AG, arabinogalactan side chains of RG-I.

Pearson correlation analyses were performed to investigate the relationship between the relative abundance of microbial genera and SCFA production at each time point (Figure 7). Seven genera (*Bifidobacterium*, *Collinsella*, *Denitrobacterium*, *Olsenella*, *Coriobacteriaceae*.1, *Bacteroidales S24-7* group.2, and *Acetitomaculum*) had significant negative correlation with propionic acid, butyric acid, valeric acid and total SCFA production. Within these, *Bacteroidales S24-7* group.2, *Olsenella*, *Coriobacteriaceae*.1, and *Acetitomaculum* also significantly negatively correlated with acetic acid. Eight genera (*Bacteroidales S24-7* group.1, *Prevotella* 9, *Faecalibacterium*, *Ruminococcaceae UCG-005*, *Ruminococcus* 2, *Selenomonas*, *Succinivibrio*, and *Succinivibrionaceae UCG*-*001*) significantly positively correlated with acetic acid, propionic acid, butyric acid, valeric acid and total SCFA production. Within these, *Ruminococcus* 2 and *Succinivibrio* also had significant positive correlation with caproic acid. *Bacteroidales S24-7* group, *Sarcina*, and *Oribacterium* had significant positive correlation with propionic acid, butyric acid, valeric acid and total SCFA production. *Roseburia*, *Ruminococcaceae NK4A214* group and *Ruminococcaceae UCG-002* significantly positively correlated with acetic acid, propionic acid, butyric acid and total SCFA production. *Prevotella* 7 significantly positively correlated with propionic acid, valeric acid, caproic acid, and total SCFA production.

The correlation between binding of mAbs and relative abundance of microbial genera was also analyzed. Within the mAbs that recognizing non-fucosylated XG, CCRC-M93 had significant negative correlation with *Bacteroidales S24-7* group.1, *Christensenellaceae R-7* group, [*Eubacterium*] *nodatum* group, *Blautia*, *Lachnospira*, *Lachnospiraceae NK3A20* group, *Roseburia*, *Ruminococcaceae UCG*-*005*, *Ruminococcus* 2, *Subdoligranulum*, *Catenibacterium*, *Catenisphaera*, *Succiniclasticum*, and *Succinivibrionaceae UCG*-*001*. CCRC-M96 significantly negatively correlated with *Bacteroidales S24-7* group.2 and *Acetitomaculum*, while it significantly positively correlated with *Anaerotruncus*. CCRC-M99 had significant negative correlation with Dorea and *Ruminococcus* 2, whereas it had significant positive correlation with *Megasphaera*. CCRC-M104 had significant positive correlation with *Prevotellaceae UCG*-*001*, while it negatively correlated with *Ruminococcaceae UCG*-*002*.

MH4.2A4 that recognizes linseed mucilage RG-I had significant negative correlations with *Bacteroidales S24-7* group.1, *Ruminococcaceae NK4A214* group, and *Ruminococcaceae UCG*-*005*.

CCRC-M105 that binds to Xylan-2 had significant negative correlations with *Prevotellaceae*.1, *Family XIII UCG*-001, *Acidaminococcus*, and *Megasphaera*, whereas it positively correlated with *Ruminococcus* 2 and *Subdoligranulum*.

CCRC-M30, binding to RG-Ic, had significant positive correlations with *Denitrobacterium*, *Olsenella*, *Bacteroidales S24-7* group.2, and *Acetitomaculum*, while it significantly negatively correlated with *Roseburia*, *Faecalibacterium*, *Ruminococcaceae UCG*-*002*, *Ruminococcaceae UCG-005*, *Selenomonas*, and *Succinivibrionaceae UCG-001*.

MAC204, that recognizing AG-1, significantly negatively correlated with *Denitrobacterium*, *Olsenella*, *Bacteroidales S24*-*7* group.2, *Acetitomaculum*, and *Roseburia*, while it had significant positive correlations with *Lactobacillus*, *Sarcina*, *Roseburia*, *Ruminococcaceae NK4A214* group, *Ruminococcaceae UCG*-*002*, *Ruminococcaceae UCG*-*005*, and *Selenomonas*.

CCRC-M133, binding to AG-2, significantly negatively correlated with *Denitrobacterium*, *Prevotella* 7, *Prevotellaceae UCG*-*001*, *Acetitomaculum*, and *Subdoligranulum*, while it positively correlated with *Ruminococcaceae UCG*-*002*.

CCRC-M131, that binds to pectic backbone, had significant negative correlation with *Denitrobacterium*, *Olsenella*, *Bacteroidales S24-7* group.2, *Prevotellaceae UCG*-*001*, *Acetitomaculum*, and *Subdoligranulum*, while it positively correlated with *Lactobacillus*, *Anaerotruncus*, and *Ruminococcaceae UCG*-*002*.

With respect to mAbs binding to AG-4, CCRC-M78 had significant negative correlation with *Bifidobacterium*, and *Christensenellaceae R-7* group, whereas it significantly positively correlated with *Prevotellaceae UCG*-*001*. CCRC-M91 had significant negative correlation with *Denitrobacterium*, *Olsenella*, and *Acetitomaculum*, and CCRC-M91 and CCRC-M92 significantly positively correlated with *Lactobacillus*. JIM13 had significant negative correlation with *Subdoligranulum*.

As for mAbs binding to RG-I/AG, they had significant negative correlation with *Bacteroidales S24-7* group (CCRC-M32), *Bacteroidales S24-7* group.2 (CCRC-M32 and M125), *Lactobacillus* (CCRC-M24), [*Eubacterium*] *nodatum* group (CCRC-M60), *Acetitomaculum* (CCRC-M42), *Blautia* (CCRC-M60), *Coprococcus* 3 (CCRC-M24), *Ruminococcaceae NK4A214* group (CCRC-M32, M41, M44, and M60), *Ruminococcaceae UCG*-002 (CCRC-M44), *Ruminococcaceae* UCG-005 (CCRC-M32, M44, and M60) *Subdoligranulum* (CCRC-M 42), and *Succiniclasticum* (CCRC-M125). Significantly positive correlations were observed with *Olsenella* (CCRC-M44), *Bacteroidales S24*-*7* group (CCRC-M128), *Prevotella* 7 (CCRC-M24 and M128), *Prevotellaceae UCG*-*001* (CCRC-M 24 and M128), *Prevotellaceae* (CCRC-M128), *Lactobacillus* (CCRC-M42), *Lachnospira* (CCRC-M128), *Ruminococcus* 2 (CCRC-M128), *Subdoligranulum* (CCRC-M128), and *Succinivibrio* (CCRC-M128).

## Discussion

### Cell Wall Polysaccharides Composition of Differently Pretreated RSM

Cell wall polysaccharides of RSM (*Brassica napus*) were comprehensively studied by chemical methods (Pustjens et al., 2013), which showed that they generally consisted of arabinan, homogalacturonan, Rhamnogalacturonan I (RG-I), type II arabinogalactan (AG), and xyloglucan (XG) (Siddiqui and Wood, 1977). In a previous study (Long et al., 2020b), we determined the monosaccharide constituent composition, which was in line with Pustjens et al. (2013). Our current findings with the mAbs are also in line with this, since non-fucosylated XG-, RG- I-, pectic backbone-, and AG-recognizing mAbs bound to CON. By similar reasoning, xylan was also detected in CON, which was not reported before, although it is not entirely clear how much cross-reactivity the mAbs show.

ALK treatment intensively increased binding of mAbs that specifically bind to non-fucosylated XG (Figure 2). This is consistent with previous finding that alkali could extract XG from hemicellulose (Pustjens et al., 2013), which led to XG being detected by the mAbs in the current study. Previous research also demonstrated that XG is linked via hydrogen bonds to the surface of cellulose microfibrils, which is extractable with alkali, but not accessible by enzyme (Pauly et al., 1999). However, enzymatic treatments (PECT1, PECT2, and CELL) led to a disappearance of binding non-fucosylated XG-specific mAbs compared to CON, which was unexpected. It could be that XG might become entrapped by other cell wall structures after pectinase or cellulase treatment. It is unknown whether this kind of the hidden phenomenon would reduce the degradability of XG.

Enzymatic treatments increased binding of linseed mucilage RG-I-specific mAbs and some mAbs directed against the arabinogalactan side chains of RG-I (RG-I/AG), and reduced binding of some mAbs directed to pectic backbone, which indicated that PECT1 and PECT2 broke down pectic backbone and exposed RG-I and its arabinogalactan side chains. These results are supported by a previous study showing that PECT1 and PECT2 degraded pectic backbone and released RG-I and its arabinogalactan side chains (Pustjens et al., 2013). Moreover, RG-I attached to cellulose microfibrils can be released by cellulase (Oechslin et al., 2003), while at the same time it might block the accessibility of pectic backbone because of the shifting of the polysaccharides structure.

ALK treatment led to the disappearance of binding of mAbs that specifically bind to AG-1 while it increased binding of mAbs that specifically binding to AG-2, which indicated that ALK could cause arabinogalactan to be physically entrapped in other cell wall structures, while linear and branched arabinan become accessible (Pattathil et al., 2010).

They were lower in amount of binding of mAbs in AG-4 from ALK_B, PECT1_B, PECT2_B, and CELL_B, compared to ALK_A, PECT1_A, PECT2_A, and CELL_A, respectively. This indicated that small fragments were produced in AG-4 group after enzymatic and chemical treating RSM, which were dialyzed out in ALK_B, PECT1_B, PECT2_B, and CELL_B during the subsequent predigestion treatment. Overall, both enzymatic and chemical processed RSM can release some polysaccharides but physically entrap or shield some others at the same time.

### Community Structure of Swine Microbiota Fed With Differently Pretreated RSM

PERMANOVA tests suggested significant differences (*P* = 0.004) in community structure between all pre-treatments and the CON group, though no significant differences were detected between the pre-treatments (Figure 3). Dietary fiber is known to have a considerable effect on gut microbiota composition (Flint, 2012; Yatsunenko et al., 2012; Cotillard et al., 2013). Glycome profiling of non-processed and processed RSM showed that polysaccharides structures were differentially shifted due to ALK, PECT1, PECT2, and CELL treatment in the current study, which were consistent with previous studies (Pustjens et al., 2012; de Vries et al., 2014). These observations might explain the specific genus changes in microbiota composition after microbiotas fed with ALK, PECT1, PECT2, and CELL compare to CON (Figure 4), despite the differential effect of the different treatments as assessed by the glycome profiling.

In order to understand which microbes were significantly influenced by dietary supplementation of processed RSM, microbial genera were compared among the five treatments (Figure 4). The relative abundances of *Ruminococcaceae NK4A214* group, *Ruminococcaceae UCG*-*002*, *Ruminococcaceae UCG*-*005*, *Roseburia*, *Bifidobacterium*, and *Christensenellaceae R-7* group were significantly increased in ALK, PECT1, PECT2, and CELL (Figure 4). Research has shown that genera of family *Ruminococcaceae* contain major (hemi)cellulolytic and pectinolytic species (Pettipher and Latham, 1979; Nyonyo et al., 2014; Hartinger et al., 2019a). Thus, microbes from family *Ruminococcaceae* play an important role in degrading (hemi)cellulose and pectin in their activity against recalcitrant fiber (Shinkai and Kobayashi, 2007; Flint et al., 2008). Previous research demonstrated that the higher the relative abundance of *Ruminococcaceae NK4A214* group, the higher the fiber degradability (Hartinger et al., 2019b). This observation was consistent with the current study, which showed that the SCFA production (which is usually used as evaluation of fiber degradability *in vitro*) was also higher in ALK, CELL, PECT1, and PECT2 compared to CON. Furthermore, our study also showed that the relative abundance of *Ruminococcaceae NK4A214* group, as well as *Ruminococcaceae UCG*-*002* and *Ruminococcaceae UCG*-*005*, significantly correlated with acetic acid, propionic acid, butyric acid, and (not surprisingly) total SCFA production. *Roseburia* is a well-known butyrate-producing bacteria (Duncan et al., 2002), and a primary degrader of β-mannans (La Rosa et al., 2019). The significant increase in abundance of this genus was accordance with the increased butyric acid production in the current study (Table 1), and increased predicted abundance of beta-mannosidases (EC.3.2.1.25) in the processed RSM groups (Figure 5). *Bifidobacterium* is suggested to be a common cross-feeding bacteria for sugar utilization (Cockburn and Koropatkin, 2016). Previous research has been shown that *Bifidobacterium bifidum* relies on the presence of a primary degrader in order to grow with either resistant starch or xylan both *in vitro* (Turroni et al., 2015) and *in vivo* (Turroni et al., 2016). It has also been reported that numerous *Bifidobacterium* species grew to higher cell densities accompanied by upregulating their respective saccharolytic pathways when grown in co-culture compared to their growth in monoculture (Milani et al., 2015). This finding was in accordance with the current study, where the relative abundance of *Bifidobacterium* was much higher than that of other genera, especially in ALK (data not shown). Alternatively, synergy existed among genera that were targeting the same substrates, possibly by specializing in degrading different motifs within the molecule. However, this might also lead to a competitive environment when the fermentation continues after 24 h. For instance, when a specific motif for a particular microbe runs out, the microbe might start to utilize another motif.”

The relative abundance of *Christensenellaceae R-7* group increased after enzymatic and chemical treatment, which was consistent with a previous study where rumen microbiota was fed with fibrinolytic enzyme-treated wheat straw (Ribeiro et al., 2020). *Christensenellaceae* plays an important role in degrading fiber (Mao et al., 2015) and producing acetic and butyric acid (Morotomi et al., 2012). All the observations above were supported by former reports that feed processed by carbohydrase enzymes could stimulate growth of specific microbes (Giraldo et al., 2007, 2008; Long et al., 2020c). It can be speculated that the pre-treatment of feed stuffs with carbohydrases causes the release of hydrolysis materials (presumably oligosaccharides), which promote the chemotactic response of specific bacteria, and stimulates their attachment to insoluble feed particles (this attachment is known as biofilm formation), thereby leading to growth of these microbes (Beauchemin et al., 2003; Giraldo et al., 2007; Ribeiro et al., 2015, 2018).

### Fiber Degradation and SCFA Production

Enzymatic and chemical treatment on RSM increased the amount of acetic acid, propionic acid, butyric acid, and thereby total SCFA production (Table 1). This observation was consistent with the predicted functional profiles related to carbohydrate metabolism, where the relative abundance of fiber breakdown and fermentation enzymes and pathways increased (Figure 5). This finding was in accordance with previous studies that addition of pectolytic enzymes improved degradability of non-starch polysaccharides (NSP) of RSM *in vitro* (Pustjens et al., 2012) and in broilers (de Vries et al., 2014; Pustjens et al., 2014). Giraldo et al. also reported that supplementation of exogenous cellulase increased SCFA production (Giraldo et al., 2007). Previous research demonstrated that supplementation of carbohydrase on feed before feeding could increase microbial protein production, ruminal cellulolytic bacterial numbers, and ruminal fibrinolytic activity. Thus, the findings above indicate that the enzymatic and chemical treatment on RSM could sufficiently open cell wall architecture (refer to Figure 2), to enable effective accessibility of NSP to bacterial degradation enzymes, and subsequently stimulate expression of microbial saccharolytic pathways.

No bindings of mAbs recognizing non-fucosylated XG were observed in lumen samples after the microbiota was fed with ALK or CON (Figure 6), which was unexpected as binding signals were seen in the substrates themselves prior to addition to SLIM (Figure 2). The hypothesis could be entertained that XG was immediately utilized by bacteria before our first sampling time point (after 30 min), or that the XG structures were unable to be recognized by the mAbs after supplementing them to lumen, due to entrapment by other molecules. Binding signals still existed at t24 for all mAbs that showed signal at the t0.5 time point, which indicated that these structures cannot be degraded any further or more fermentation time was needed. Glycome profiling showed that binding of mAbs recognizing each polysaccharide structure/epitope were dynamic during the 24 h fermentation period (Figure 6). It is not unlikely that certain polysaccharide structure, such as (hemi)cellulose and pectin, were exposed to microbes stage by stage, due to ever increasing degradation of the cell wall structures over time. For instance, bacteria should break down side-chain AG before they can utilize RG-I. However, this hypothesis should be validated in future study.

The increased in abundance of *Ruminococcaceae NK4A214* group, *Ruminococcaceae UCG*-*002*, and *Ruminococcaceae UCG*-*005* in enzymatic and chemical treated RSM groups showed negative correlations with XG, RG-I, and RG-I/AG (arabinogalactan side chains of RG-I), and positively correlated with AG-1, AG-2, and pectic backbone. These observations suggested that ALK, CELL, PECT1, and PECT2 stimulated these genera to utilize XG, RG-I, and arabinogalactan side chains of RG-I, and exposed AG-1, AG-2, and pectic backbone to other bacteria in the current study. The abundance of *Roseburia* and *Succinivibrionaceae UCG-001* in enzymatic and chemical treated RSM groups had negative correlations with XG and AG-1, which suggested that the treatments on RSM stimulated these genera to degrade XG and AG-1. A possible model of action can be explained by the adhesion theory of Cellulolytic Bacteria (Miron et al., 2001), which supposes that the (in our case) enzymatic and chemical treatments on RSM stimulate attachments of microbes to specific polysaccharides structures (Meale et al., 2014), which lead the bacteria to degrade them. However, the mechanism of exogenous enzymes enhanced degradation of plant cell walls is complex, with many interrelated factors, and requires further studies. Moreover, degradation of fibers requires a plethora of microbial enzymes as indicated for instance by the numerous PUL-loci needed by *Bacteroides thetaiotaomicron* to breakdown pectin.

## Conclusion

The present study clearly demonstrated that both enzymatic and chemical pre-treatment on RSM shifted its cell wall polysaccharide structure, subsequently altering microbial community composition and functional profile compared to untreated RSM, and eventually increased fiber degradability as evaluated by SCFA production. Furthermore, glycome profiling showed that the abundance of cell wall polysaccharides were dynamically changed during fermentation, and did not continuously decrease during the fermentation period. Our findings that ALK, CELL, PECT1 and PECT2 increased fiber degradability in RSM could help guide feed additive strategies to improve efficiency and productivity in swine industry. The current study gave insight into how feed enzyme modulate microbial status, which provides good opportunity to develop novel carbohydrase, particularly in swine feed.

## Data Availability Statement

The datasets presented in this study can be found in online repositories. The names of the repository/repositories and accession number(s) can be found in the article/Supplementary Material.

## Author Contributions

KV and CL designed and planned the experiment. CL performed the data analyses and drafted the manuscript. KV revised and contributed to the manuscript. Both authors read and approved the final manuscript.

## Conflict of Interest

The authors declare that the research was conducted in the absence of any commercial or financial relationships that could be construed as a potential conflict of interest.

## Footnotes

1 https://github.com/qiime2/q2-diversity

2 http://www.carbosource.net/

3 https://www.r-project.org/

## Abbreviations

RSM: rapeseed meal
CON: control RSM without treatment
CELL: cellulase Accellerase 1000 RSM treated with CELL
PECT: pectinase
PECT1, Pectinex Ultra SP: RSM treated with PECT1
PECT2, Multifect Pectinase: RSM treated with PECT2
ALK: alkaline 6 M NaOH RSM treated with ALK
_B: for carbohydrase- or ALK-treatment prior to digestion
_A: for carbohydrase- or ALK-treatment after digestion
SCFA: short chain fatty acids
NSP: non-starch polysaccharides
GIT: gastrointestinal tract
SLIM: Large Intestine in intro Model
GES: gastric electrolyte concentrate solution
TIM-2: TNO (gastro-) Intestinal Model of colon
SIEMP: standard ileal efflux medium of pigs
mAbs: Monoclonal antibodies
BSA: bovine serum albumin
TMB: tetramethylbenzidine
ASV: amplicon sequence variant
XG: xyloglucan
RG-I: Rhamnogalacturonan I
AG: arabinogalactan
RG-I/AG: arabinogalactan side chains of RG-I.
