# Supplementary material for: Pretreatment of Rapeseed Meal Increases Its Recalcitrant Fiber Fermentation and Alters the Microbial Community in an in vitro Model of Swine Large Intestine
Source: Front Microbiol. 2020 Nov 19;11:588264. doi: 10.3389/fmicb.2020.588264 (PMC7711092; doi:10.3389/fmicb.2020.588264)
Supplement: Supplementary file 1 [file Data_Sheet_1.pdf]

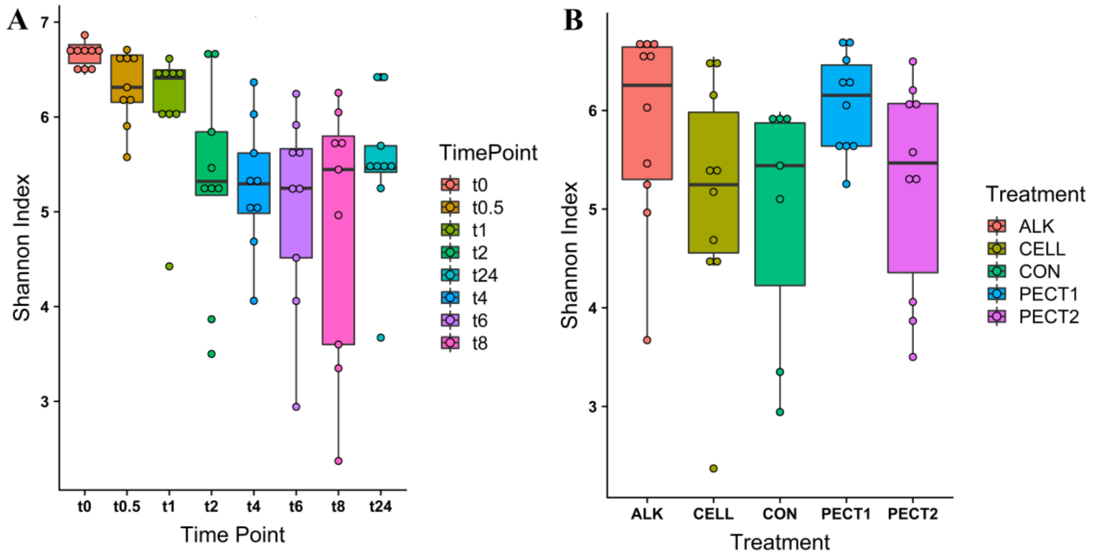

Figure S1. Community diversity represented by Shannon index at ASV level for samples from each time point (A) and treatment (B). ** $P < 0.01$.

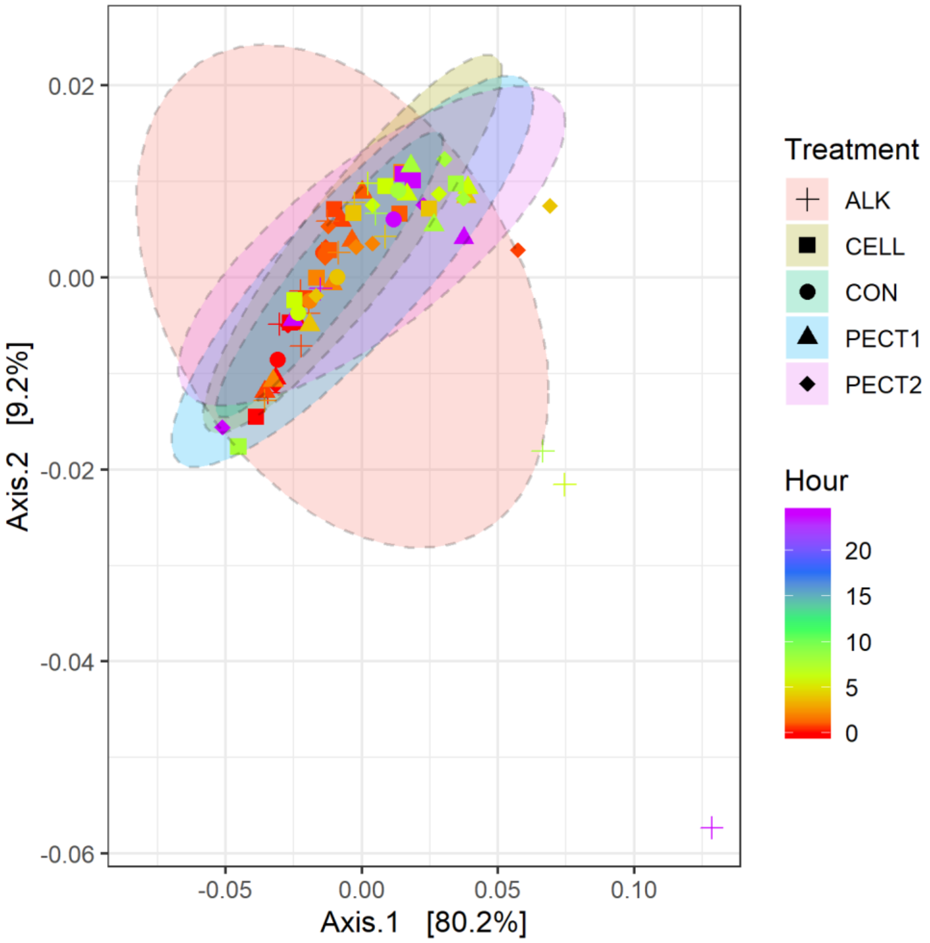

Figure S2. Principal coordinate analysis (PCoA) plot generated based on the calculated distances in weighted UniFrac matrix. Samples were grouped by shape and color in terms of treatment and time point they belonged to, respectively: CON (sphere), ALK

(plus), PECT1 (triangle), PECT2 (diamond), CELL (square); a red-green-purple scale was used to indicate the fermentation time (red and purple depict start and end of the fermentation period).

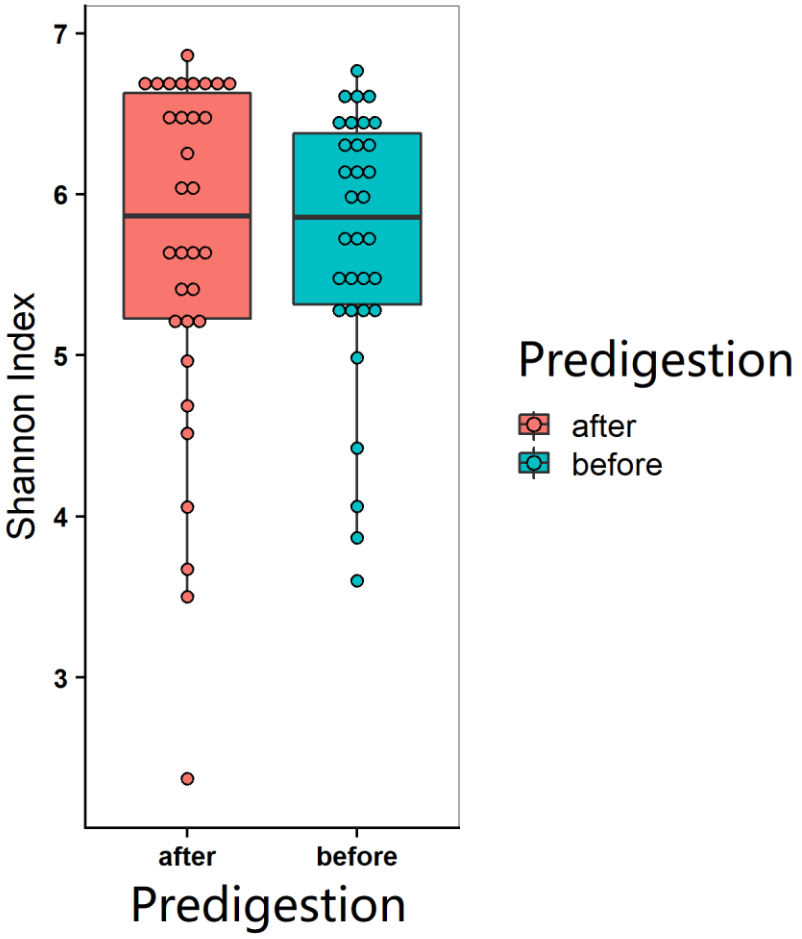

Figure S3. Community diversity represented by Shannon index at ASV level for samples from before and after predigestion.
